# Association of *PTPRT* mutations with immune checkpoint inhibitors response and outcome in melanoma and non‐small cell lung cancer

**DOI:** 10.1002/cam4.4472

**Published:** 2021-12-04

**Authors:** Wenjing Zhang, Fuyan Shi, Yujia Kong, Yuting Li, Chao Sheng, Suzhen Wang, Qinghua Wang

**Affiliations:** ^1^ Department of Health Statistics Key Laboratory of Medicine and Health of Shandong Province School of Public Health Weifang Medical University Weifang China; ^2^ Tianjin Cancer Institute, National Clinical Research Center for Cancer, Key Laboratory of Cancer Prevention and Therapy of Tianjin Tianjin Medical University Cancer Institute and Hospital Tianjin China; ^3^ Department of Epidemiology and Biostatistics, National Clinical Research Center for Cancer, Key Laboratory of Molecular Cancer Epidemiology of Tianjin Tianjin Medical University Cancer Institute and Hospital Tianjin China

**Keywords:** biomarker, immunotherapy, melanoma, NSCLC, *PTPRT* mutation

## Abstract

**Purpose:**

Protein tyrosine phosphatase receptor type T (PTPRT), which is a well‐known phosphatase and mutates frequently in melanoma and non‐small cell lung cancer (NSCLC). Our research aims to elucidate its mutation association with immune checkpoint inhibitors (ICI) efficacy.

**Methods:**

We integrated whole‐exome sequencing (WES)**‐**based somatic mutation profiles and clinical characteristics of 631 melanoma samples received ICI agents from eight studies and 109 NSCLC samples from two studies. For validation, 321 melanoma and 350 NSCLC immunotherapy samples with targeted next‐generation sequencing (NGS) were employed. Besides, an independent NSCLC cohort contained 240 samples was also collected for further corroboration. Distinct immune infiltration was evaluated according to the *PTPRT* mutational status.

**Results:**

In the WES melanoma cohort, patients with *PTPRT* mutations harbored a significantly elevated ICI response rate (40.5% vs. 28.6%, *p* = 0.036) and a prolonged survival outcome (35.3 vs. 24.9 months, *p* = 0.006). In the WES NSCLC cohort, the favorable response and immunotherapy survival were also observed in *PTPRT*‐mutated patients (*p *= 0.036 and 0.019, respectively). For the validation cohorts, the associations of *PTRPT* mutations with better prognoses were identified in melanoma, NSCLC, and pan‐cancer patients with targeted‐NGS (all *p *< 0.05). Moreover, immunology analyses showed the higher mutation burden, increased lymphocyte infiltration, decreased‐ activated‐stroma, and immune response pathways were detected in patients with *PTPRT* mutations.

**Conclusion:**

Our investigation indicates that *PTPRT* mutations may be considered as a potential indicator for assessing ICI efficacy in melanoma and NSCLC, even across multiple cancers. Further prospective validation cohorts are warranted.

## Graphical abstract

Our study suggests that PTPRT mutations may serve as a strong biomarker to assess ICI efficacy in melanoma and NSCLC, even across multiple cancers.

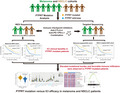

## 1 INTRODUCTION

The survival outcome of advanced or metastatic cancer patients has been markedly prolonged owing to the emergence of immune checkpoint inhibitors (ICI). The ICI treatments have become the first‐line selection for several cancers, such as melanoma and non‐small cell lung cancer (NSCLC).^1^, ^2^, ^3^ Nevertheless, only a fraction of patients exhibited the clinical benefits of ICI agents. The three FDA‐approved immunotherapy efficacy indicators, including programmed‐death ligand‐1 (PD‐L1) expression,^3^ microsatellite instability (MSI),^4^ and tumor mutation burden (TMB)^5^ exhibit remarkable effects in clinical practice. However, they are sometimes ineffective in evaluating ICI responses.

The protein tyrosine phosphatase receptor type T (PTPRT), which contains 37 exons and spans over 500 kilobases, is a member of the PTP family. Recent two studies provided in vivo and in vitro evidence and demonstrated that PTPRT normally acts as a cancer suppressive mediator in colon tumor.^6^, ^7^ In addition, *PTPRT* has been determined as a significantly mutated gene (SMG) in lung squamous cell carcinoma,^8^ colorectal cancer,^9^ and melanoma.^10^ Chen et al. reported that *PTPRT* mutations may be associated with gastric cancer metastasis.^11^ Another study involved in lung adenocarcinoma revealed that the African American patients had a markedly elevated *PTPRT* mutations than the European American patients and suggested the clinical significance for the recruitment of the minority population in clinical trials.^12^ Based on the mutational profile of The Cancer Genome Atlas (TCGA) derived from the cBioPortal, the alteration rate of *PTPRT* was 29.9% in melanoma and >10% in patients with lung adenocarcinoma, stomach, colorectal, uterine, and esophageal cancers.^13^

Recent studies have demonstrated that mutations in single genes, such as *POLE*,^14^ *PBRM1*,^15^ *TTN*,^16^ *MUC16*,^17^ *B2M*,^18^ *JAK1*/*2* ^19^, ^20^ were linked with ICI response or resistance. Specific mutational signatures, for example, tobacco smoking, APOBEC, and ultraviolet light exposure‐related signatures were frequently accumulated in ICI responders.^21^ An activated‐stroma signature was identified to exhibit immune‐suppressive roles in cancer immunity.^22^ The well‐known epithelial to mesenchymal transition (EMT) signal was also associated with tumor immune escape.^23^, ^24^

*PTPRT* is frequently mutated in tumors; nevertheless, its connection with ICI efficacy was incompletely elucidated. In this study, we first integrated ICI‐treated melanoma and NSCLC samples with whole‐exome sequencing to explore the link between *PTPRT* mutation and ICI survival. Then, melanoma and NSCLC samples with targeted next‐generation sequencing were also curated for further validation. Via multiple verifications, we here suggest the solid connection between *PTPRT* mutation and immunotherapy effect.

## 2 MATERIALS AND METHODS

### 2.1 Melanoma and NSCLC samples collection and study design

Whole‐exome sequencing (WES) based somatic mutation data of 631 melanoma patients received immune checkpoint inhibitors (ICI) agents (i.e., anti‐CTLA‐4, anti‐PD‐l/PD‐L1, or combination) were acquired from previous eight studies,^20^, ^25^, ^26^, ^27^, ^28^, ^29^, ^30^, ^31^ and 109 NSCLC patients were from two studies.^32^, ^33^ We used the Oncotator to uniformly re‐annotate all somatic mutations curated in this study.^34^ Non‐synonymous alterations were used for analyses. The predicted MHC binding affinity scores and HLA types, which were used for evaluating neoantigen counts were curated from 224 melanoma^25^, ^27^, ^30^ and 109 NSCLC samples.^32^, ^33^ The detailed sequencing and clinical characteristics, including age, sex, stage, ICI treatment information, and so on are illustrated in Table S1 for melanoma and Table S2 for NSCLC. A patient was considered to be efficacious to ICI treatment if the response status was complete response (CR) or partial response (PR).

A total of 1661 ICI‐treated pan‐cancer patients, who underwent the Integrated Mutation Profiling of Actionable Cancer Targets (MSK‐IMPACT) assay of a targeted 468‐gene next‐generation sequencing (NGS) at Memorial Sloan Kettering Cancer Center (MSKCC) were collected.^35^ Among, 321 were melanoma patients and 350 were NSCLC. The detailed clinical and treatment information are shown in Table S3. Besides, another independent NSCLC cohort^36^ contained 240 samples with also targeted‐NGS was employed for further corroboration (Table S4).

**FIGURE 1:**
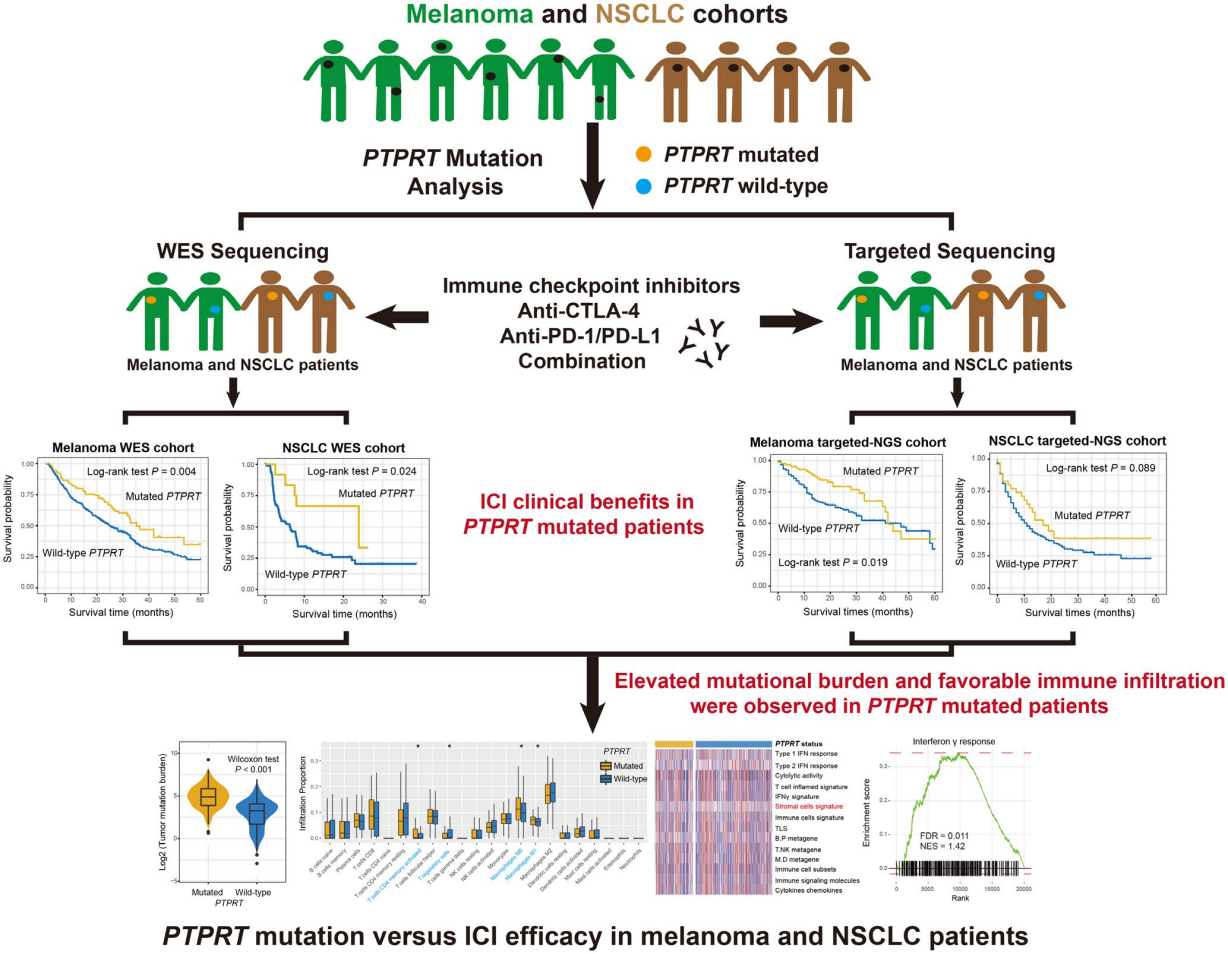
The flow chart of this work. Graphical abstract exhibition of the association of *PTPRT* mutations with immune checkpoint inhibitors efficacy and immune‐related factors in melanoma and NSCLC samples with distinct sequencing methods

Gene expression, somatic mutation profiles, and clinical characteristics of 457 melanoma and 995 NSCLC samples derived from the TCGA were acquired from Genome Data Commons (https://gdc.cancer.gov). In this work, all mRNA expression‐related analyses were achieved by using the gene expression data from the TCGA. The workflow of this study is provided in Figure 1.

### 2.2 Deciphering mutational signatures operative in the genome

The method proposed by Kim et al.^37^ was employed to detect mutational signatures from integrated melanoma and NSCLC cohorts. The core of this algorithm is Bayesian variant nonnegative matrix factorization (NMF). Specifically, NMF was employed to decompose mutation portrait matrix *A* that contained the 96 base substitution classes. Matrix A was divided into two nonnegative matrices *W* and *H* (i.e., *A *≈ *WH*), where *W* indicates the detected mutational signatures and *H* represents the mutation activities of each corresponding signature. The column of matrix *A* is the count of detected signatures and rows representing the 96 base substitution types. The rows and columns of matrix *H* indicate the individual signatures and their corresponding mutational activities, respectively. All extracted mutational signatures were subsequently annotated with the 30 well‐curated signatures stored in the COSMIC database (version 2) based on cosine similarity.

### 2.3 Evaluation of tumor infiltration immune cells

CIBERSORT algorithm was applied to infer the tumor infiltration proportion of 22 immune cell types based on the LM22 signature.^38^ Angelova et al. established an 812‐immune‐metagene signature to infer 31 distinct immune cells infiltration and tumor immune landscape,^39^ specific genes for each assessed immune cell are curated in Table S5. We used both methods to obtain comprehensive immune infiltration results.

### 2.4 Microenvironment‐based immune‐related signatures

Previously reported immune‐related signatures were collected as follows: (1) immune and stromal cells signatures, which respectively reflect the total immune and stromal cell infiltration levels in microenvironment^40^; (2) immune cell subsets, enrichment of T cells, B cells, and NK cells^41^; (3) T/NK, B/P, and M/D metagene, which respectively indicate the activities of T/NK cells, B/plasma cells, and monocytes/dendritic cells^42^; (4) Type 1/2 IFN response, which are two distinct interferon response types functioned respectively by interferon α and γ^43^; (5) IFNγ signature, which plays vital roles in the immune response and ICI efficacy^44^; (6) T cell‐inflamed signature, a factor associated with IFNγ response^45^; (7) immune cytolytic activity^43^; (8) immune signaling molecules^41^; (9) cytokines and chemokines^41^; (10) TLS, which is tertiary lymphoid structures associated with inflammation response.^46^ The detailed feature genes for each immune signature are shown in Table S6.

### 2.5 A signature of activated‐stroma

Moffitt et al. reported a stroma‐related signature,^22^ which was defined by two distinct features (i.e., activated‐stroma and normal‐stroma). Based on the nearest template prediction (NTP) algorithm^47^ with distinct feature gene subgroups, the activated stromal subtype could be identified.

### 2.6 GSVA and GSEA

Single sample gene set enrichment analysis (ssGSEA) method within GSVA package^36^ was applied to infer the enrichment scores of all curated immune signatures for each sample based on the specific feature genes. Differential analysis of gene expression profile according to *PTPRT* mutation status was achieved with R package DESeq2.^37^ The *t* values extracted from differential results were then employed to performed gene set enrichment analysis (GSEA) implemented by fgsea package (https://github.com/ctlab/fgsea). The well‐annotated pathways in hallmark gene sets and KEGG from Molecular Signatures Database (MSigDB) were used as the background signals. The false discovery rate (FDR) and normalized enrichment score (NES) were obtained based on 1 million permutations.

### 2.7 Association of *PTPRT* mutations with mutational burden

Genome instability is always influenced by alterations in genomic maintenance regulators.^48^ Therefore, multivariate logistic regression models were performed with mutations in genomic maintenance genes (i.e., *BRCA1*/*2*, *TP53*, and *POLE*) and detected mutational signatures taken into consideration to obtain an adjusted association between *PTPRT* mutations and mutational burden. In our work, TMB was calculated as the log2 transformation of total non‐synonymous mutations per megabase in both WES and TCGA cohorts; for the targeted cohorts, TMB was acquired from the supplementary file. The neoantigen burden (NB) for 224 melanoma and 109 NSCLC WES samples was evaluated according to a recent method provide by Balachandran et al.^49^ The neoantigen data of 340 melanoma and 656 NSCLC samples from the TCGA cohort were downloaded from the Cancer Immunome Atlas (TCIA, https://www.tcia.at/home).

### 2.8 Statistical analyses

R software (version 4.0.2) was used to complete related calculations. Mutational patterns for specific genes were illustrated with maftools package.^50^ Heatmap representation of distinct subgroups was achieved based on pheatmap package. Survival curves were obtained by using the Kaplan–Meier approach and the Log‐rank test to analyze the differences. Multivariate Cox regression models embedded in forestmodel package were employed to control confounding variables and obtain the adjusted results. Correlation of continuous and categorical variables with *PTPRT* mutational status was assessed with Wilcoxon rank‐sum test and Fisher exact test, respectively. Two‐sided *p* values less than 0.05 were considered to be statistically significant.

## 3 RESULTS

### 3.1 *PTPRT* mutations in WES melanoma cohort

**FIGURE 2:**
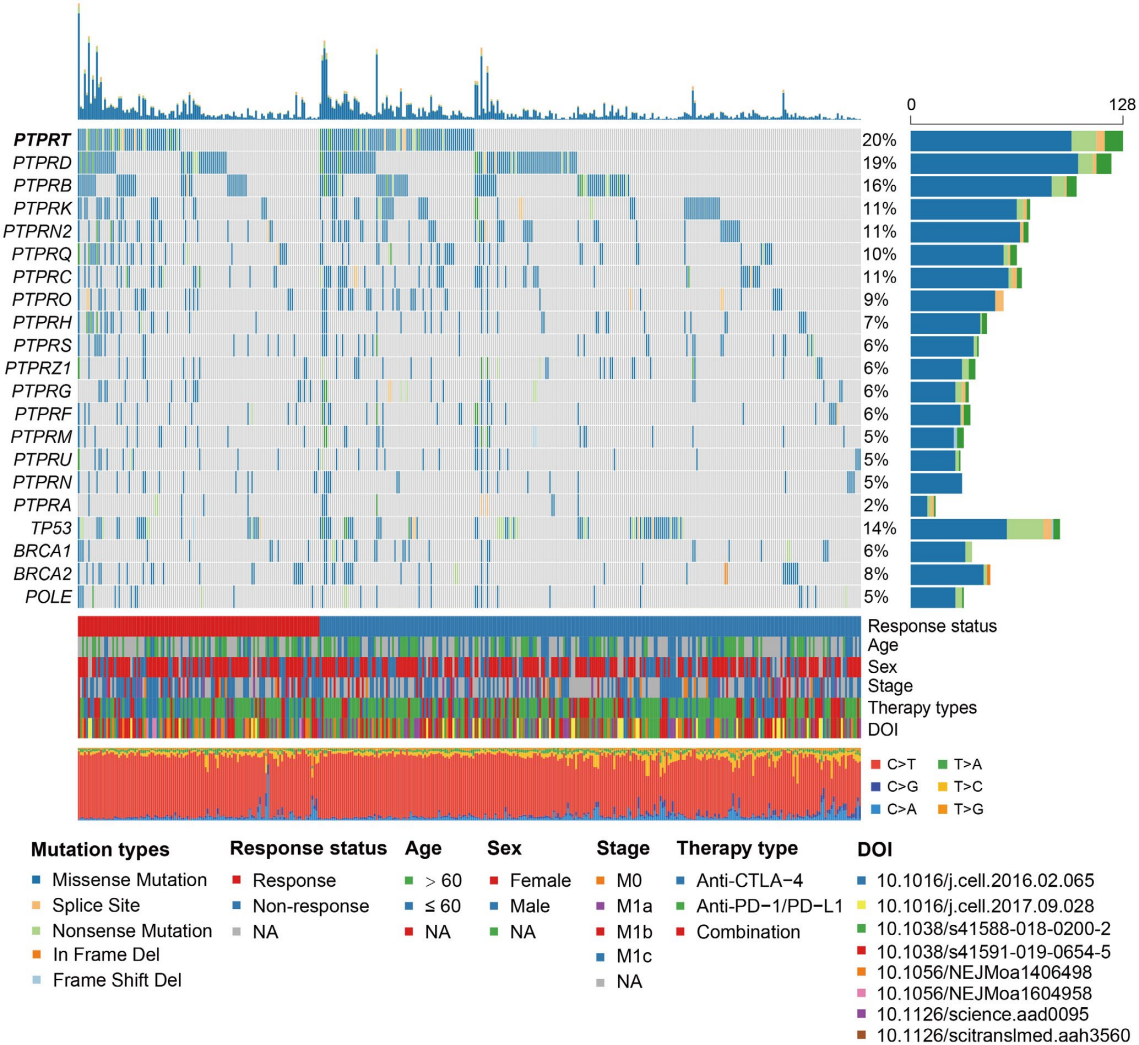
Waterfall plot representation of the mutational patterns of *PTPRT*, its family members, and genomic integrity maintenance genes. The left panel shows the gene symbols, the upper panel indicates the non‐synonymous mutation counts for each sample, the middle plot illustrates mutational patterns of the included genes with distinct mutation types colored distinctly, the right penal shows the mutation rate of each gene, and the bottom panel indicates immunotherapy response status, clinical characteristics, and base substitution categories

Of the 631 melanoma samples derived from eight WES immunotherapy studies, 193 (30.6%) were recognized as the ICI treatment responders. This integrated melanoma cohort was dominated by C > T mutations (Figure 2). The PTP family members and genome integrity maintenance genes (e.g., *BRCA1*, *BRCA2*, *TP53*, and *POLE*) with respect to *PTPRT* mutations are exhibited in the Figure 2. We observed that *PTPRT* was the most frequently mutated gene in the PTP family, contributing to 126 of 631 melanoma samples (20.0%). Detailed amino acid changes of *PTPRT* mutations are shown with the lollipop plot (Figure S1).

### 3.2 *PTPRT* mutations predictive of melanoma immunotherapy outcome and response

**FIGURE 3:**
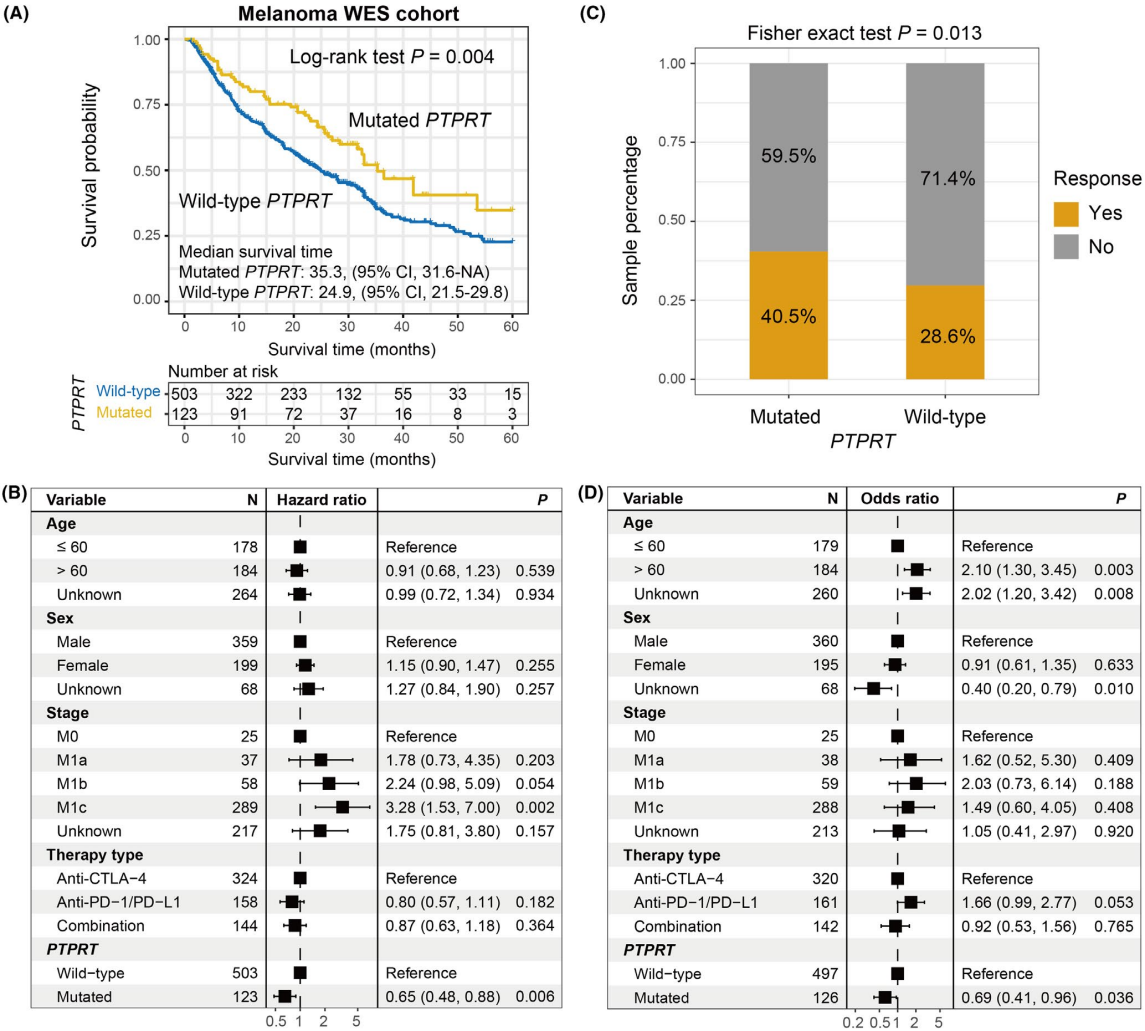
*PTPRT* mutations predictive of ICI efficacy in the aggregated melanoma WES cohort. (A) Kaplan–Meier curves of the distinct *PTPRT* status. (B) Forest plot representation of the connection between *PTPRT* mutations and ICI prognosis with clinical confounding factors taken into consideration. (C) Fisher exact test and (D) multivariate logistic regression model evaluation of *PTPRT* mutations versus ICI response rate

Results demonstrated that patients with *PTPRT* mutations harbored a significantly prolonged ICI prognosis as compared with those without such mutations (median survival time: 35.3 vs. 24.9 months, Log‐rank test *p* = 0.004; Figure 3A). This link was still existing when controlling for age, sex, stage, and therapy type in the multivariate Cox regression analysis (HR: 0.65, 95% CI: 0.48–0.88, *p *= 0.006; Figure 3B). Prognostic abilities of *PTPRT* mutations in the individual cohort and distinct treatment types were calculated and relevant results were shown as Figures S2 and S3, respectively. We observed that *PTPRT* mutations were also connected with the elevated immunotherapy response rate (40.5% vs. 28.6%, Fisher exact test *p *= 0.013; Figure 3C). Multivariate logistic model was conducted with clinical confounders taken into consideration and the result still reached the statistical significance (OR: 0.69, 95% CI: 0.41–0.96, *p *= 0.036; Figure 3D).

### 3.3 *PTPRT* mutations versus mutational burden in melanoma

**FIGURE 4:**
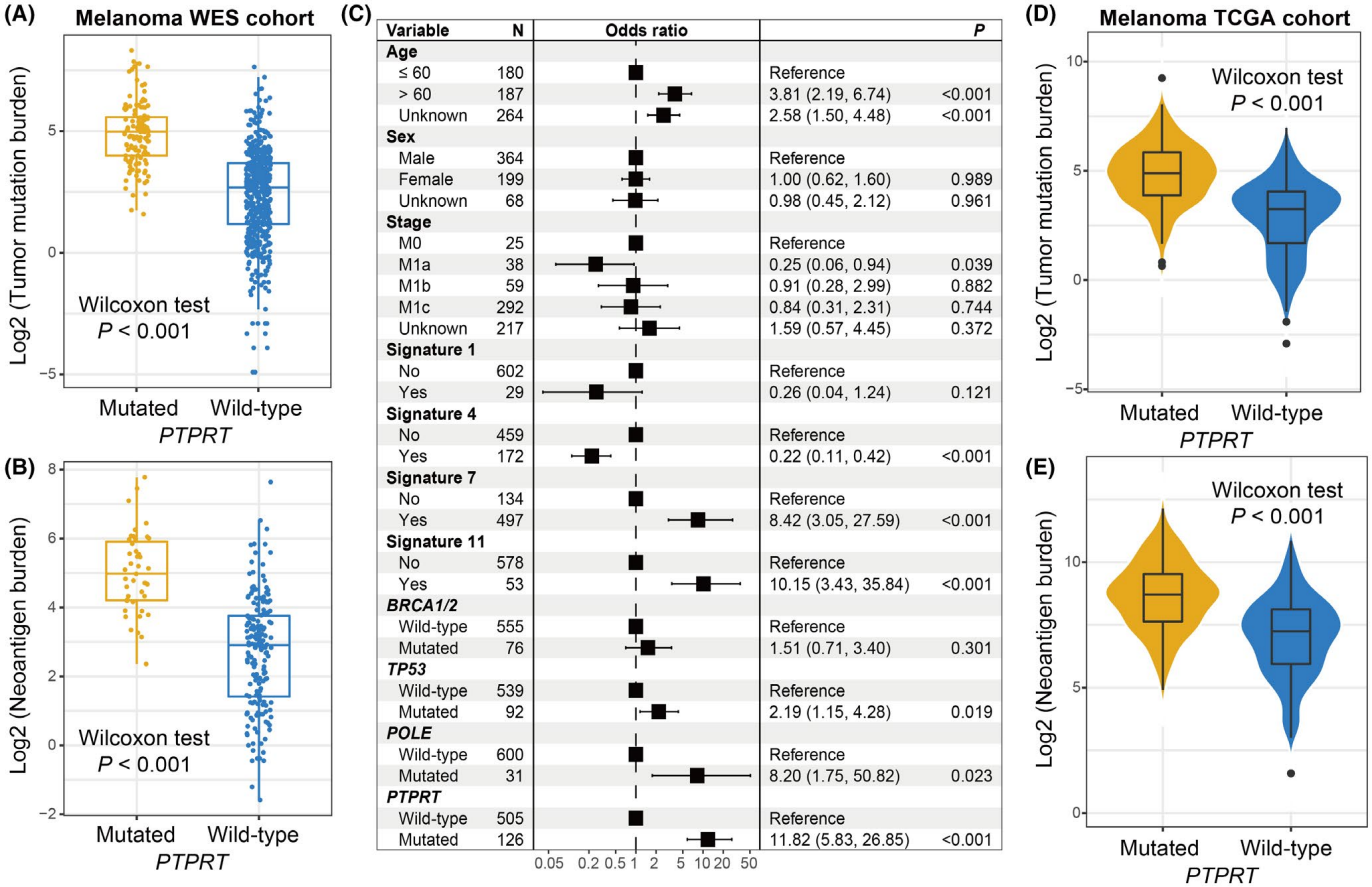
*PTPRT* mutations association with mutational burden in melanoma. Distribution of (A) TMB and (B) NB in distinct *PTPRT* subgroups in the pooled melanoma WES cohort. (C) Multivariate regression model underlying the association between *PTPRT* mutations and TMB with clinical features, extracted mutational signatures, and genome repair gene mutations taken into account to adjust confounders. Distribution of (D) TMB and (E) NB in distinct *PTPRT* subgroups in the TCGA melanoma cohort

The markedly higher TMB and NB were found in melanoma patients with *PTPRT* mutations (median TMB: 4.99 vs. 2.61, median NB: 5.01 vs. 2.98, both *p *< 0.001; Figure 4A,B). Since specific mutational signatures operative in the genome could result in genomic instability and variational mutation rates. We extracted four mutational signatures from this pooled melanoma cohort by annotating with COSMIC (Figure S4A); they are age‐related signature 1, smoking‐related signature 4, ultraviolet light exposure‐induced signature 7, and alkylating agent treatment‐induced signature 11 (Figure S4B). The extracted detailed mutational activities for melanoma cohort are exhibited in Table S7. To eliminate the probability that the connection between *PTPRT* mutations and TMB was influenced by other miscellaneous variables, we incorporated clinical factors, detected signatures (i.e., 1, 4, 7, and 11), and mutations of *BRCA1*/*2*, *TP53*, and *POLE* into the multivariate logistic analysis. Association of *PTPRT* mutations with TMB was still significative after adjusted analysis (OR: 11.82, 95% CI: 5.83–26.85, *p *< 0.001; Figure 4C). The consistent results of *PTPRT* mutations with elevated TMB and NB were also obtained based on the melanoma samples from the TCGA cohort (both *p* < 0.001; Figure 4D,E).

### 3.4 *PTPRT* mutations association with ICI efficacy and mutational burden in WES NSCLC cohort

**FIGURE 5:**
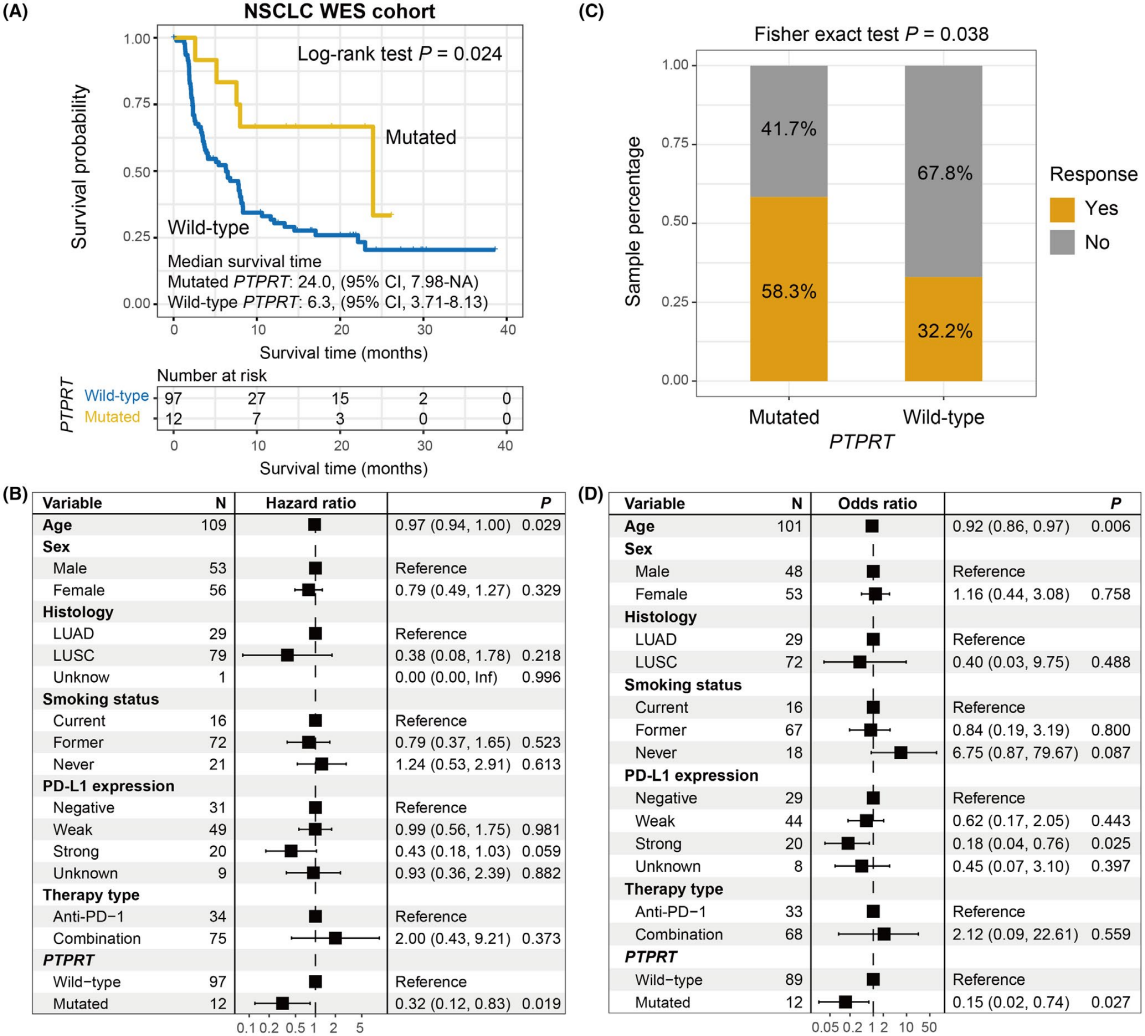
*PTPRT* mutations predictive of ICI efficacy in the aggregated NSCLC WES cohort. (A) Kaplan–Meier curves of the distinct *PTPRT* status. (B) Forest plot representation of the connection between *PTPRT* mutations and ICI prognosis with clinical confounding factors taken into consideration. (C) Fisher exact test and (D) multivariate logistic regression model evaluation of *PTPRT* mutations versus ICI response rate

Of the 109 curated NSCLC WES samples, 36 (33.0%) were evaluated as the immunotherapy responders. *PTPRT* also mutated frequently in NSCLC, accounting for 12 of 109 patients (11.0%). Via the Kaplan–Meier survival analysis, we demonstrated that *PTPRT*‐mutated NSCLC patients exhibited a preferable ICI survival outcome than those wild‐type patients (median survival time: 24.0 vs. 6.3 months, Log‐rank test *p *= 0.024; Figure 5A). This link remained stable in the multivariate‐adjusted Cox model with confounding factors (i.e., age, sex, histology, smoking status, PD‐L1 expression, and therapy type) incorporated (HR: 0.32, 95% CI: 0.12–0.83, *p *= 0.019; Figure 5B). Prognosis analyses of *PTPRT* mutations in distinct cohorts and treatment types are illustrated in Figure S5. The further exploration showed that an enhanced ICI response rate was observed in patients with *PTPRT* mutations (58.3% vs. 32.2%, Fisher exact test *p* = 0.038; Figure 5C); and this link was still significant even adjusted for multiple confounders (OR: 0.15, 95% CI: 0.02–0.74, *p* = 0.027; Figure 5D).

**FIGURE 6:**
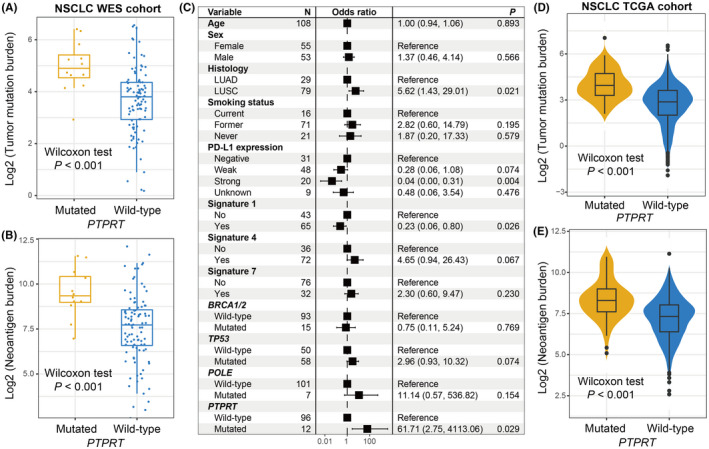
*PTPRT* mutations association with mutational burden in NSCLC. Distribution of (A) TMB and (B) NB in distinct *PTPRT* subgroups in the pooled NSCLC WES cohort. (C) Multivariate regression model underlying the association between *PTPRT* mutations and TMB with clinical features, extracted mutational signatures, and genome repair gene mutations taken into account to adjust confounders. Distribution of (D) TMB and (E) NB in distinct *PTPRT* subgroups in the TCGA NSCLC cohort

In this WES NSCLC cohort, the markedly increased TMB and NB were found in *PTPRT* mutant patients (median TMB: 4.91 vs. 3.84, median NB: 9.27 vs. 7.73, both *p* < 0.001; Figure 6A,B). We extracted three mutational signatures from NSCLC patients (Table S8). In the multivariate logistic regression model, we included clinical variables, extracted signatures, and DNA repair gene mutations; and the connection between *PTPRT* mutations and higher TMB was still existing (OR: 61.71, 95% CI: 2.75–4113.06, *p* = 0.029; Figure 6C). Based on the NSCLC samples from TCGA, we also noticed the positive associations of *PTPRT* mutations with TMB and NB (both *p* < 0.001; Figure 6D,E).

### 3.5 Corroboration in melanoma and NSCLC patients with targeted‐NGS

**FIGURE 7:**
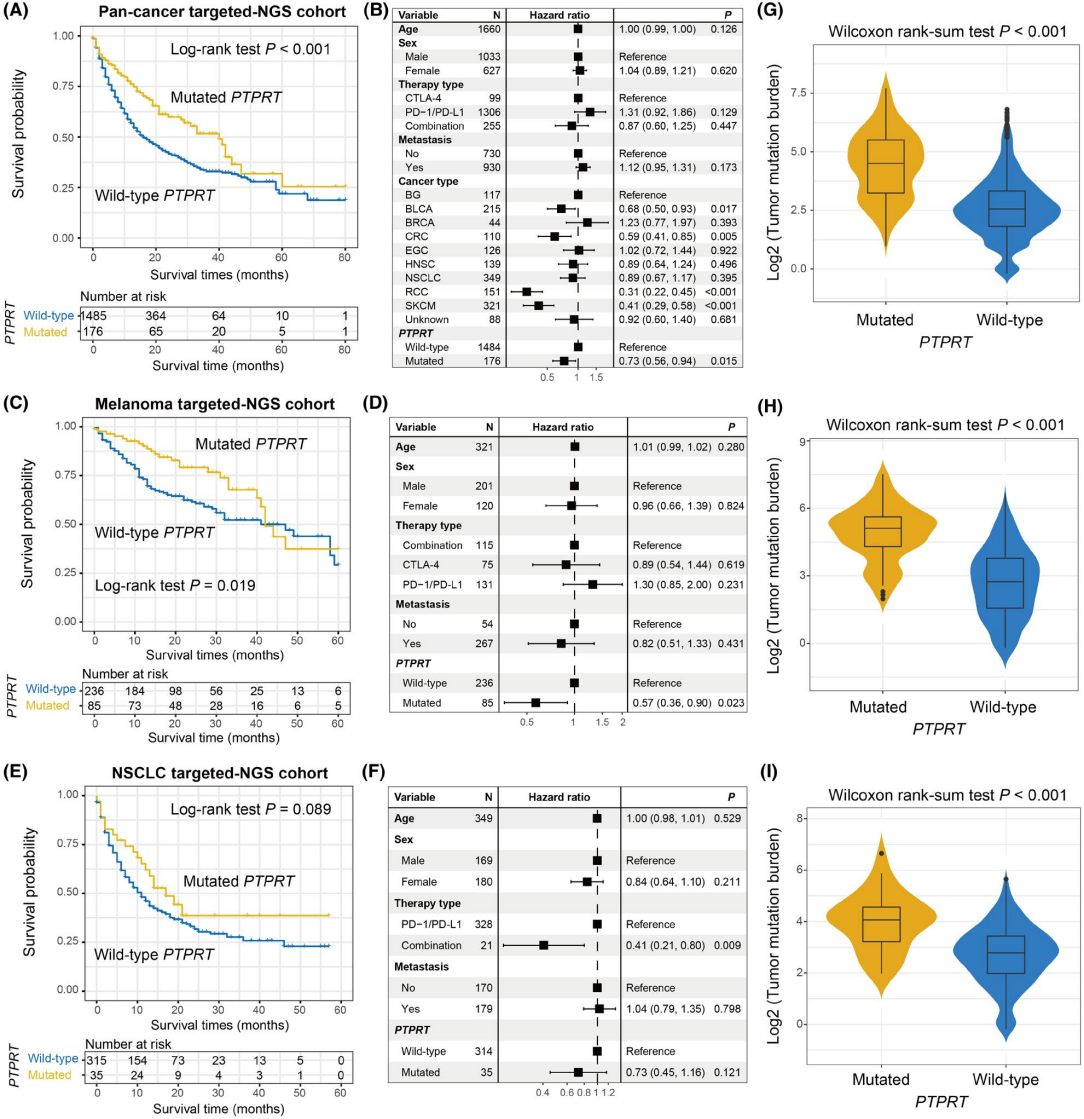
Validation of the link of *PTPRT* mutations with preferable ICI prognosis and elevated TMB with targeted‐NGS cohorts. Kaplan–Meier survival curves of distinct *PTPRT* groups and adjusted forest plots illustration in (A, B) pan‐cancer, (C, D) melanoma, and (E, F) NSCLC samples, respectively. Box plots showed the connections between *PTPRT* mutations and TMB in (G) pan‐cancer, (H) melanoma, and (I) NSCLC samples, respectively

To validate the immunotherapy implications of *PTPRT* mutations, we employed 1661 ICI‐treated pan‐cancer patients with targeted‐NGS. Survival analysis showed that *PTPRT* mutations were linked with the favorable survival outcome across multiple cancers (Log‐rank test *p* < 0.001; Figure 7A). This link still reached statistical significance after adjusting age, sex, therapy type, metastasis status, and cancer type (HR: 0.73, 95%CI: 0.56–0.94, *p* = 0.015; Figure 7B). We subsequently evaluated *PRPRT* mutations versus prognosis in melanoma and NSCLC patients derived from this targeted pan‐cancer cohort. Accordant with aforementioned results, melanoma patients with *PTPRT* mutations harbored a markedly preferable ICI prognosis than those without such mutations in Kaplan–Meier analysis (Log‐rank test *p *= 0.019; Figure 7C) and multivariate Cox model (HR: 0.57, 95% CI: 0.36–0.90, *p* = 0.023; Figure 7D). For the NSCLC, a marginally significantly better prognosis was observed in *PTPRT* mutant subgroup in both univariate (Log‐rank test *p* = 0.089; Figure 7E) and multivariate analyses (HR: 0.73, 95% CI: 0.45–1.16, *p *= 0.121; Figure 7F). The elevated TMB of *PTPRT* mutations was also observed in pan‐cancer, melanoma, and NSCLC cohorts (all *p* < 0.001; Figure 7G–I).

### 3.6 Further corroboration in an independent NSCLC cohort with targeted‐NGS

**FIGURE 8:**
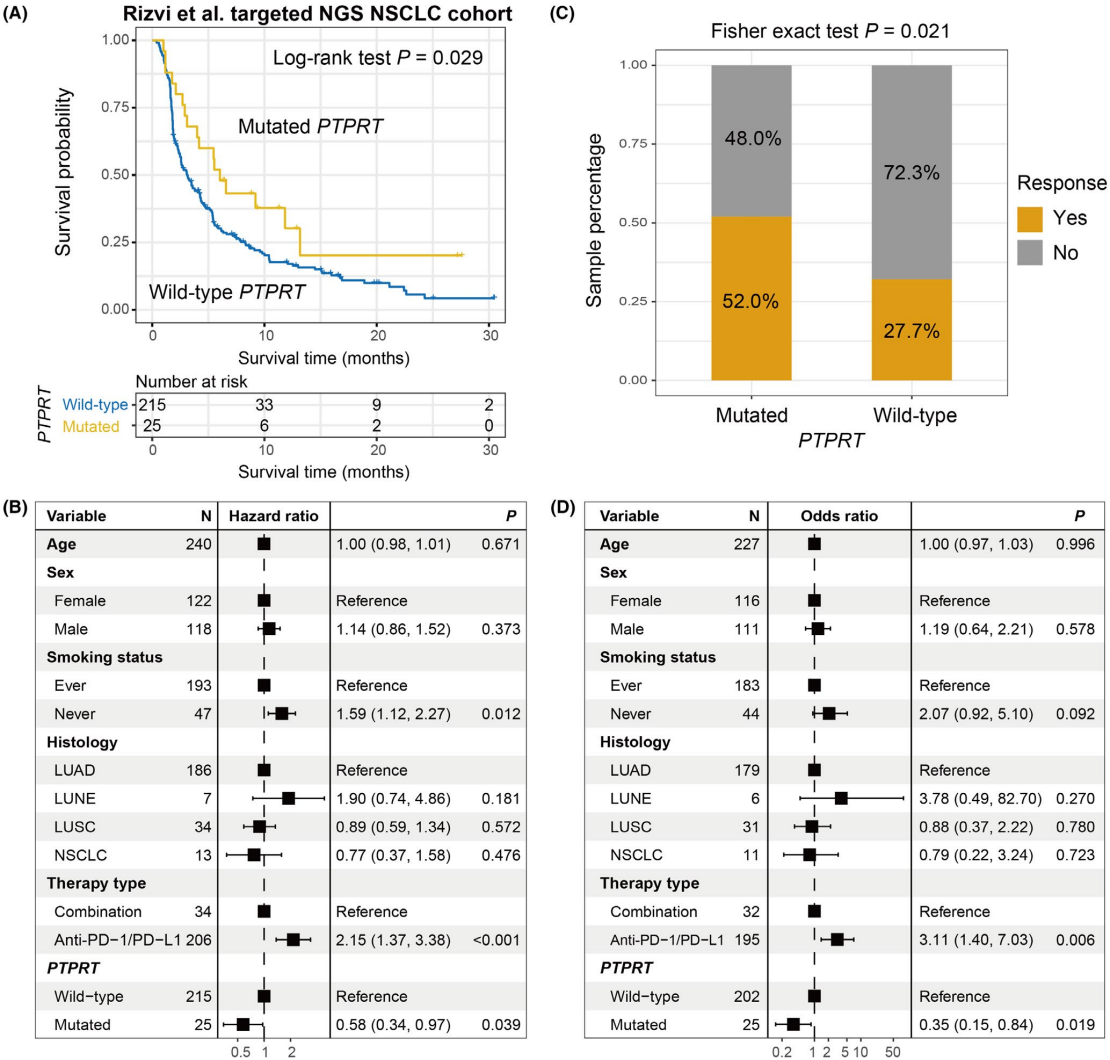
Further corroboration with an independent targeted NSCLC cohort. (A) Kaplan–Meier curves of the distinct *PTPRT* status. (B) Forest plot representation of the connection between *PTPRT* mutations and ICI prognosis with clinical confounding factors taken into consideration. (C) Fisher exact test and (D) multivariate logistic regression model evaluation of *PTPRT* mutations versus ICI response rate

From Rizvi et al. study,^36^ we obtained 240 NSCLC samples who underwent targeted sequencing and ICI treatments. We further validated the prognosis roles of *PTPRT* mutations in this independent cohort. As expected, *PTPRT* mutations are connected with a favorable ICI survival (Log‐rank test *p* = 0.029; Figure 8A), and this association was still significant in multivariate‐adjusted model (HR: 0.58, 95% CI: 0.34–0.97, *p* = 0.039; Figure 8B). In addition, the increased immunotherapy response rate was also noticed in patients with *PTPRT* mutations via Fisher exact test (52.0% vs. 27.7%, *p* = 0.021; Figure 8C) and logistic regression model (OR: 0.35, 95% CI: 0.15–0.84, *p *= 0.019; Figure 8D). *PTPRT* mutant group also had a markedly higher TMB than wild‐type group in this cohort (Wilcoxon rank‐sum test *p *< 0.001; Figure S6).

### 3.7 **Immunocyte infiltration, immune‐relevant signatures, and pathways associated with** *PTPRT* **mutations**

**FIGURE 9:**
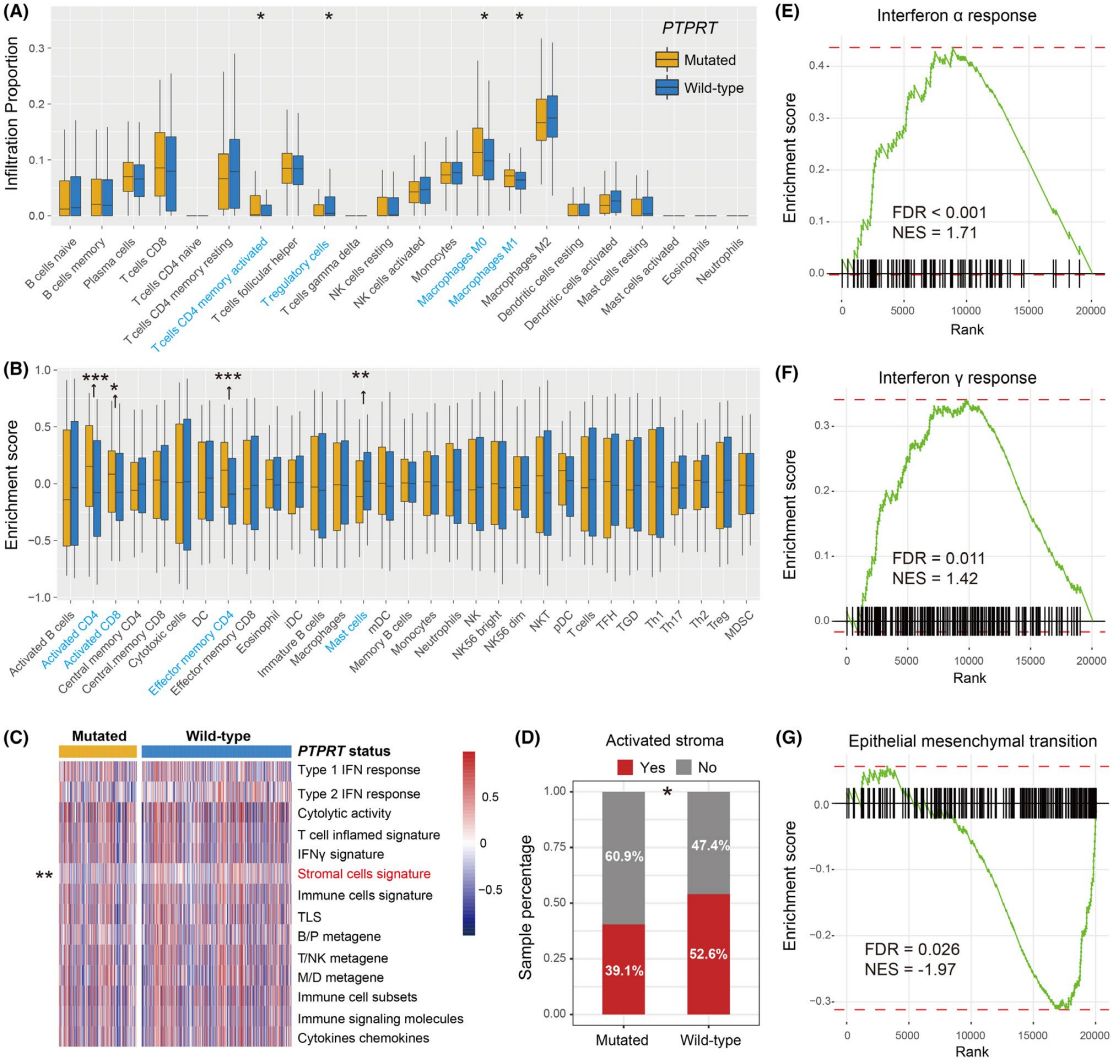
Immunology analyses of *PTPRT* mutations in melanoma. The abundance of distinct immune infiltration cells was calculated with (A) CIBERSORT and (B) Angelova et al. methods, separately. Significantly differentially infiltrated immune cells between two subgroups were highlighted with blue. (C) Heatmap illustrated the enrichment scores of immune‐related signatures in *PTPRT* mutated versus wild‐type patients. The significantly differentially enriched immune signature between two subgroups was highlighted with red. (D) Distinct distribution proportion of activated‐stroma subtype in *PTPRT* mutated versus wild‐type patients. Enrichment of the immune‐relevant pathways, such as (E) Interferon α and (F) Interferon γ responses in the *PTPRT*‐mutated group. (G) Enrichment of the immune‐suppressive EMT signaling pathway in the *PTPRT* wild‐type group. * *p* < 0.05; ** *p* < 0.01; *** *p* < 0.001

Considering the crucial roles of *PTPRT* mutations for immunotherapy prognosis evaluation, we explored the potential mechanisms behind *PTPRT* mutations in melanoma. Immune cell analysis showed that more infiltration of immune‐responsive cells (i.e., activated CD4 and CD8 T cells, effector memory CD4 T cells, and M1 macrophages) and lesser infiltration of immune‐suppressive cells (i.e., regulatory T cells) were connected with *PTPRT* mutations (Wilcoxon rank‐sum test, all *p *< 0.05; Figure 9A,B). Besides, we also observed the decreased abundance of mast cells (*p *= 0.002; Figure 9B), which were previously revealed as an immune inhibitor.^51^, ^52^ The ssGSEA analysis against gene expression profile indicated that of 14 immune signatures, the stromal cell signature enrichment was negatively associated with *PTPRT* mutations (*p* = 0.006; Figure 9C). Moreover, a reduced proportion of activated‐stroma, which plays immune‐suppressive roles, was simultaneously observed in the *PTPRT* mutant group (Fisher exact test *p* = 0.039; Figure 9D). GSEA pathway results (Figure S7) suggested that two interferon‐mediated immune signals, including IFNα and IFNγ responses, were both enriched in the top circuits of patients with *PTPRT* mutations (NES = 1.71 and 1.42, respectively; both FDR <0.05; Figure 9E,F). Consistently, the well‐known EMT pathway, which promotes tumor immune escape, was absent in the *PTPRT* mutant group (NES = −1.97, FDR = 0.026; Figure 9G).

In NSCLC, the enhanced infiltration of CD8 T cells and M1 macrophages (both *p* < 0.05), reduced infiltration of M2 macrophages (*p* = 0.026), and immune response‐relevant circuits (e.g., antigen processing and presentation, graft‐versus‐host disease, and allograft rejection) were also observed in *PTPRT*‐mutated patients (Figure S8A,B).

## 4 DISCUSSION

We conducted an integrative immunotherapy analysis of *PTPRT* mutations in melanoma and NSCLC patients with both WES and targeted sequencing. Results indicated that *PTPRT* mutations were linked with the prolonged ICI survival outcome and response rate, which may be attributed to the more favorable immune infiltration and enhanced mutational burden. A strength of this work is that our observations were cross‐validated with distinct cancer types and sequencing platforms. These findings demonstrate that *PTPRT* mutations may be considered as a strong indicator for evaluating immunotherapy effect in melanoma, NSCLC, even across multiple cancers.

Besides melanoma and NSCLC, *PTPRT* was also frequently mutated in other several cancers, such as stomach, colorectal, uterine, and esophageal cancers, as described by the cBioPortal TCGA data. *PTPRT* was always not considered as the SMG candidate due to its large size. Two similar genes were *TTN* and *MUC16*, but recent studies have revealed their mutations were strongly correlated with the favorable prognosis or immunotherapy outcome,^16^, ^53^ suggesting their potential roles for immunotherapy evaluation. Mechanisms under the connection between *PTPRT* mutation and immune response are not fully elucidated. A leading explanation indicates that PTPRT plays phosphorylation functions involved in the JAK‐STAT pathway,^54^, ^55^ which is a critical mediator in T cell immunity,^56^ PD‐1 signal,^57^ and antigen presentation.^58^

Recently numerous studies have been revealed that PTPs played vital roles in the immune regulation. PTPRA was reported to regulate the lymphocyte function via the modulation of oncogenic FYN signaling.^59^ PTPRC is a key point of T/B cell antigen receptor activation in leukemia and lymphoma.^60^ Aberrations of *PTPRD* were identified to implicate in chronic lymphocytic leukemia,^61^ and PTPRD was demonstrated as the tumor suppressor in hepatocellular carcinoma by regulating the PD‐1/PD‐L1 axis.^62^ In lung adenocarcinoma, the elevated metastasis ability and decreased NK cell activity were identified to be associated with PTPRN overexpression.^63^ PTPRZ was recognized as a novel immunotherapy target in glioblastoma owing to its multiple roles in immune surveillance.^64^

Recent two studies also revealed *PTPRT* mutations may be implicated in immunotherapy response.^65^, ^66^ He et al.^65^ used only one aggregated tumor cohort to explore the potential connection of *PTPRT* mutations and did not conduct the multivariate‐adjusted analyses, these may introduce biases to the final results. Wang et al.^66^ performed the relevant exploration only for NSCLC patients and lacked additional cancer type validation. In our study, by using multiple distinct cohorts and across‐validation under distinct sequencing platforms, we could obtain a reliable association between *PTPRT* mutations and ICI outcomes.

A study demonstrated that *PTPRT* may predict bevacizumab chemotherapy resistance with deleterious mutation of *PTPRT* causing a poor prognosis in metastatic colorectal cancer.^55^ Based on the results from TCGA melanoma and NSCLC cohorts, patients with *PTPRT* mutations did not exhibit the clinical benefits of simply chemotherapy (Figure S9A,B), but had the favorable treatment prognosis in patients treated with immune checkpoint‐based agents, suggesting the specific predictive roles of *PTPRT* mutations in the immunotherapy settings.

Recently several studies have demonstrated the vital roles of mutations in a single gene for assessing ICI efficacy. Jia et al. observed that *TTN* mutations were positively linked with ICI determinants and immunotherapy survival outcome in melanoma and NSCLC.^16^ Patients with *POLE*/*POLD1* mutations harbored a markedly favorable prognosis in a pan‐cancer ICI cohort contained 1644 patients.^14^ In metastatic renal cell carcinoma patients received nivolumab antibody, Braun et al. found that preferable overall and progression‐free survival were significantly associated with *PBRM1* mutations.^15^ The high TMB is a promising indicator in cancer immunotherapy, nevertheless, some factors, such as uncertain threshold, exome sequencing fees, and bias of distinct platforms largely influence the accurate assessment of the TMB.^16^ Instead of performing WES sequencing and determining a certain threshold, *PTPRT* mutational status could be obtained by using the targeted sequencing methods, which will reduce the sequencing fee and make the TMB evaluation and ICI prognosis prediction more easily. Therefore, *PTPRT* mutations may be an alternative surrogate for predicting ICI response in melanoma and NSCLC.

This work employed public cohorts of distinct institutions, which may introduce some biases in the procedures of data integration and analysis. In addition, gene expression‐related findings were calculated based on the TCGA cohorts, rather than the initial ICI‐treated dataset, which may incompletely illuminate the mechanisms of ICI outcome. As a result, the links between *PTPRT* mutation pattern and distinct immunology, including analysis of lymphocyte infiltration, immune‐related signatures and oncogenic pathways, needs further experimental verification.

## 5 CONCLUSION

In summary, in this integrative study, *PTPRT* mutation was identified as a putative strong biomarker to infer immune checkpoint‐based treatment responses in melanoma, NSCLC, even across multiple cancers. Relevant results were obtained under mutual validation with distinct cancer types and sequencing platforms. Further prospective verification cohorts and mechanistic studies are needed.

## CONFLICT OF INTEREST

The authors declare that they have no conflict of interest.

## AUTHORS CONTRIBUTION

QW and SW designed this study; QW, SW, WZ, and FS collected and integrated the related genomic data; WZ, FS, QW, SW, YK, YL, and CS conducted distinct data analysis; WZ, FS, QW, and SW composed and corrected the manuscript.

## ETHICS APPROVAL AND CONSENT TO PARTICIPATE

All samples used in this study were obtained from the previously published datasets and the informed consent has been completed.

## Supporting information

Fig S1‐S9

Table S1‐S8

## Data Availability

All samples used in this work are acquired from the previously published studies.
