# Supplementary material for: Association of PTPRT mutations with immune checkpoint inhibitors response and outcome in melanoma and non‐small cell lung cancer
Source: Cancer Med. 2021 Dec 4;11(3):676–91. doi: 10.1002/cam4.4472 (PMC8817076; doi:10.1002/cam4.4472)
Supplement: Supplementary file 1 — Fig S1‐S9 [file CAM4-11-676-s001.docx]

**Su
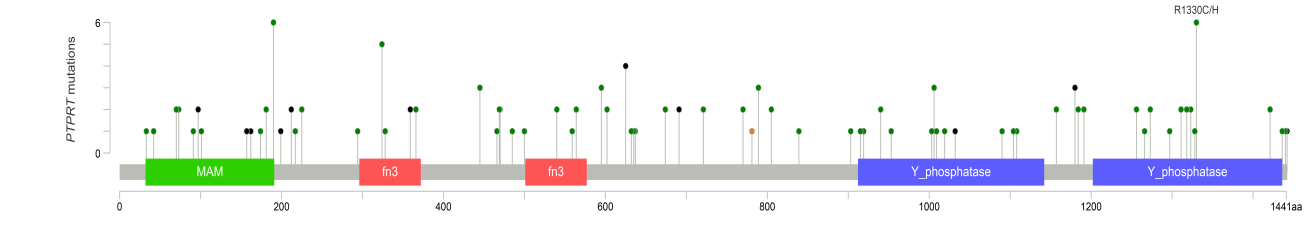
pplementary Figures**

**Figure S1.** Lollipop plot exhibition of the detailed amino acid changes of *PTPRT* mutations in the WES melanoma cohort.

**
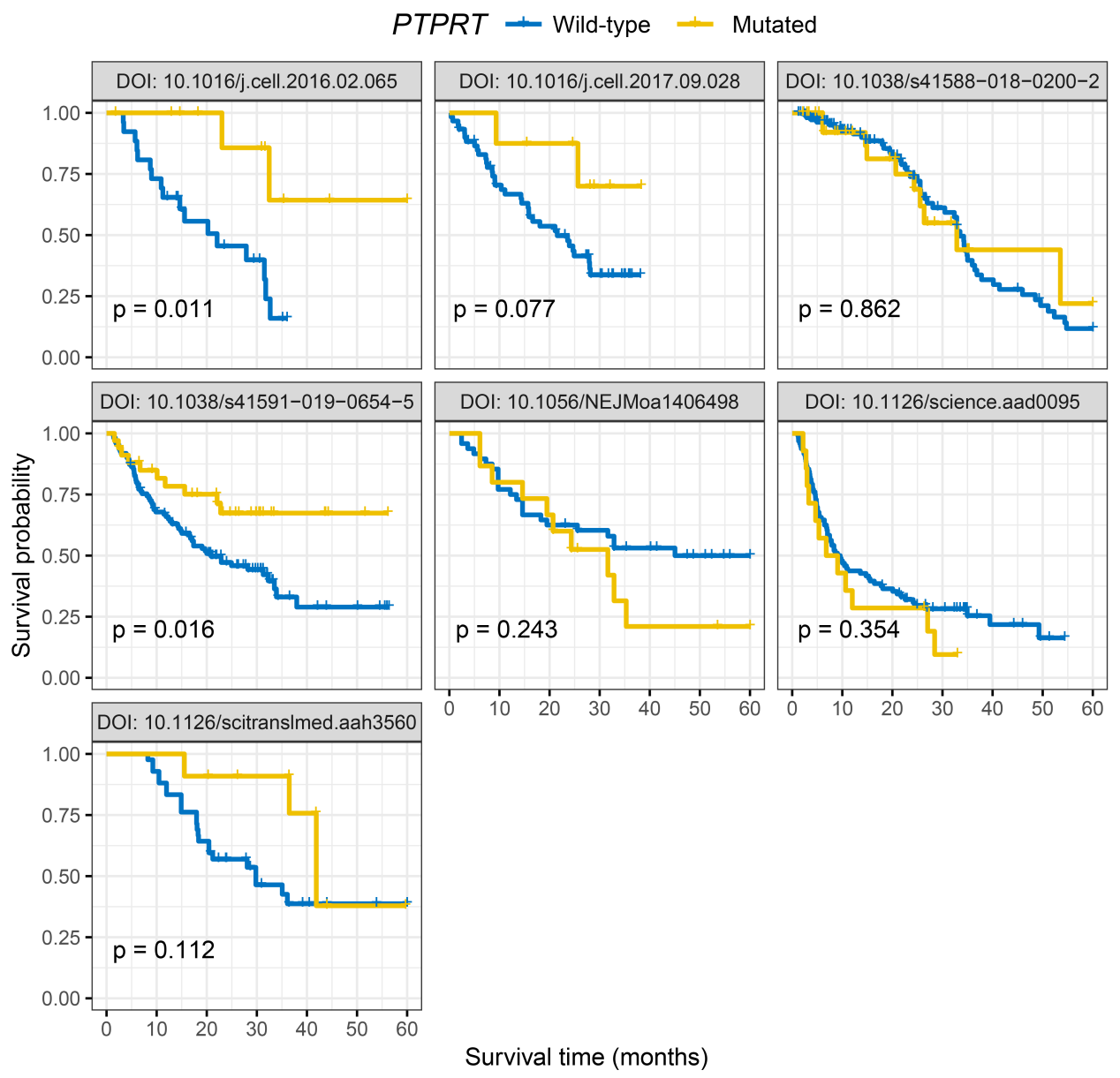
**

**Figure S2.** Kaplan-Meier survival curves stratified by the *PTPRT* mutational status in each WES melanoma cohort.

**
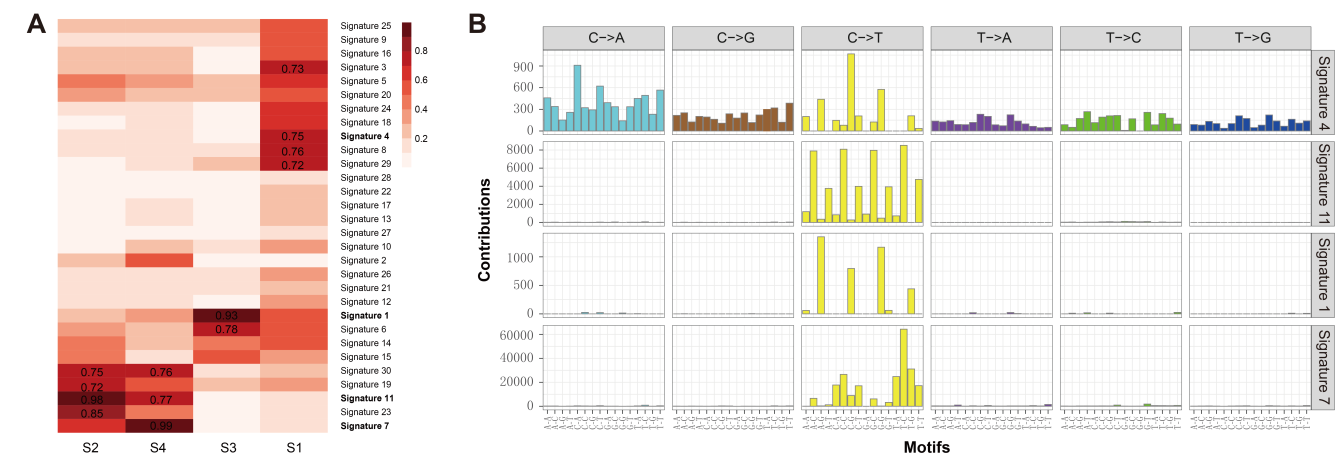

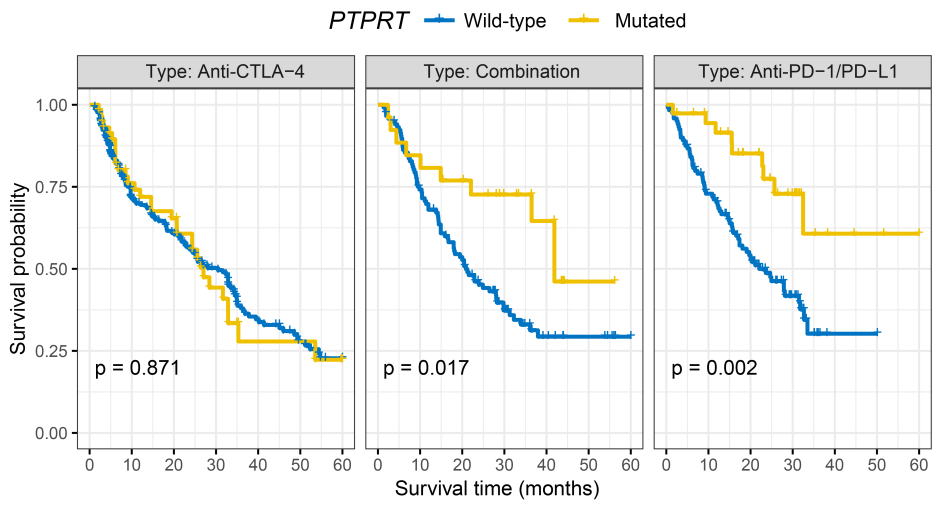
Figure S3.** Kaplan-Meier survival curves stratified by the *PTPRT* mutational status under distinct immunotherapy types in melanoma.

**Figure S4.** Extraction of the 4 mutational signatures in melanoma. (A) Cosine similarity between extracted mutational signatures and the 30 well-annotated signatures from the COSMIC. (B) Identification of the 4 mutational signatures according to the COSMIC nomenclature and their mutational patterns illustration.

**
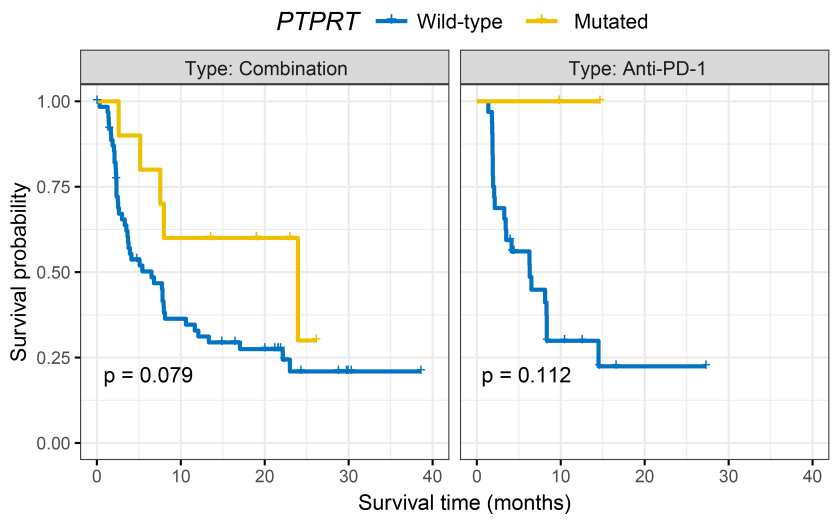
Figure S5.** Prognosis analyses of *PTPRT* mutations in individual NSCLC cohort.

**Figure S6.** A**
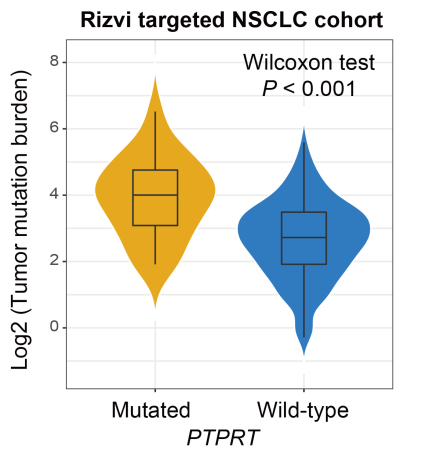
**ssociation of *PTPRT* mutations with tumor mutational burden in Rizvi *et al.* cohort illustrated by the box plot.

**
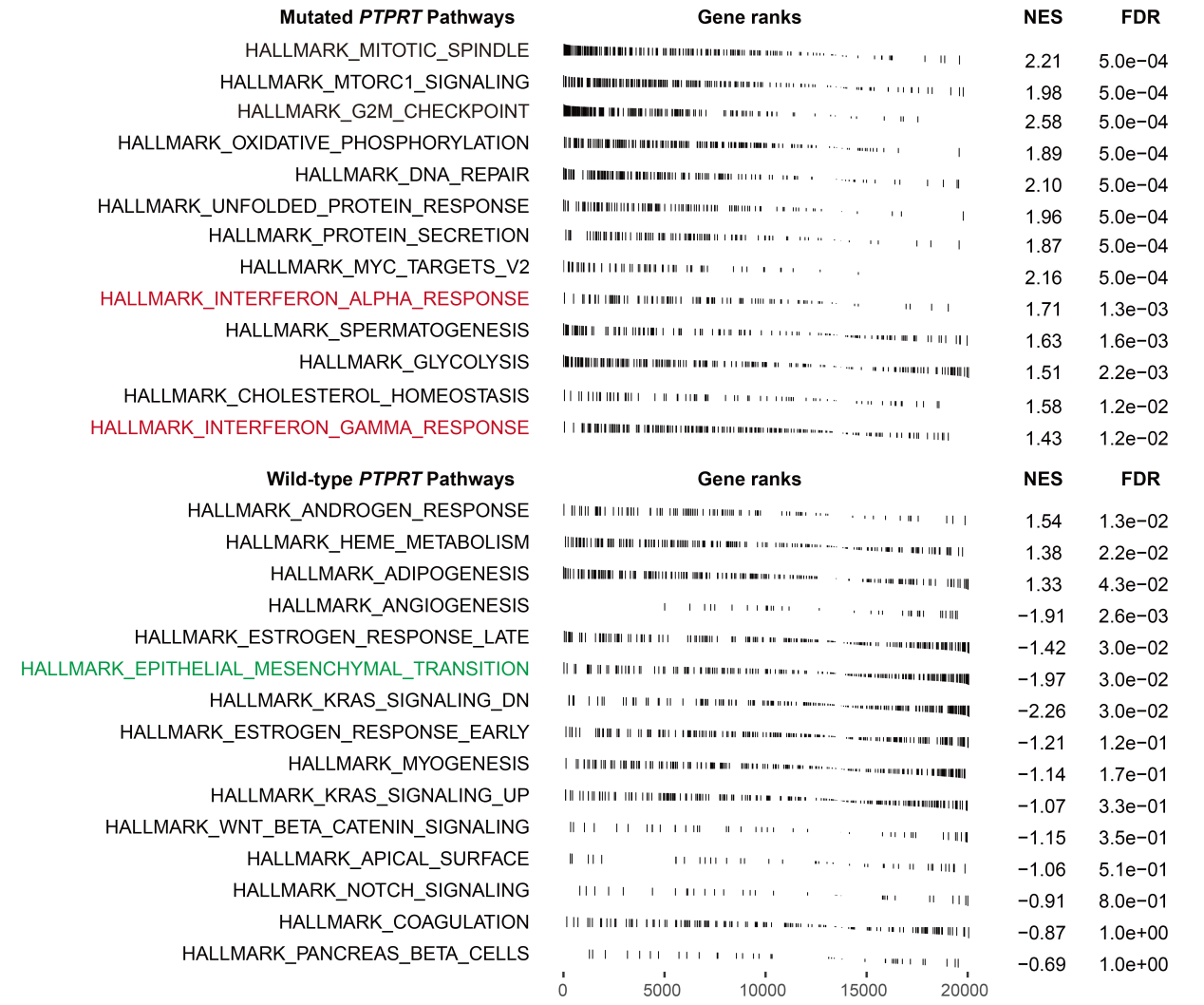
**

**Figure S7.** Significantly enriched top signaling pathways in *PTPRT* mutant and wild-type subgroups in melanoma. Pathways indicated by red were pro-inflammatory signals in *PTPRT* mutated patients, whereas indicating by green was immune inhibition signals in *PTPRT* wild-type patients.

**
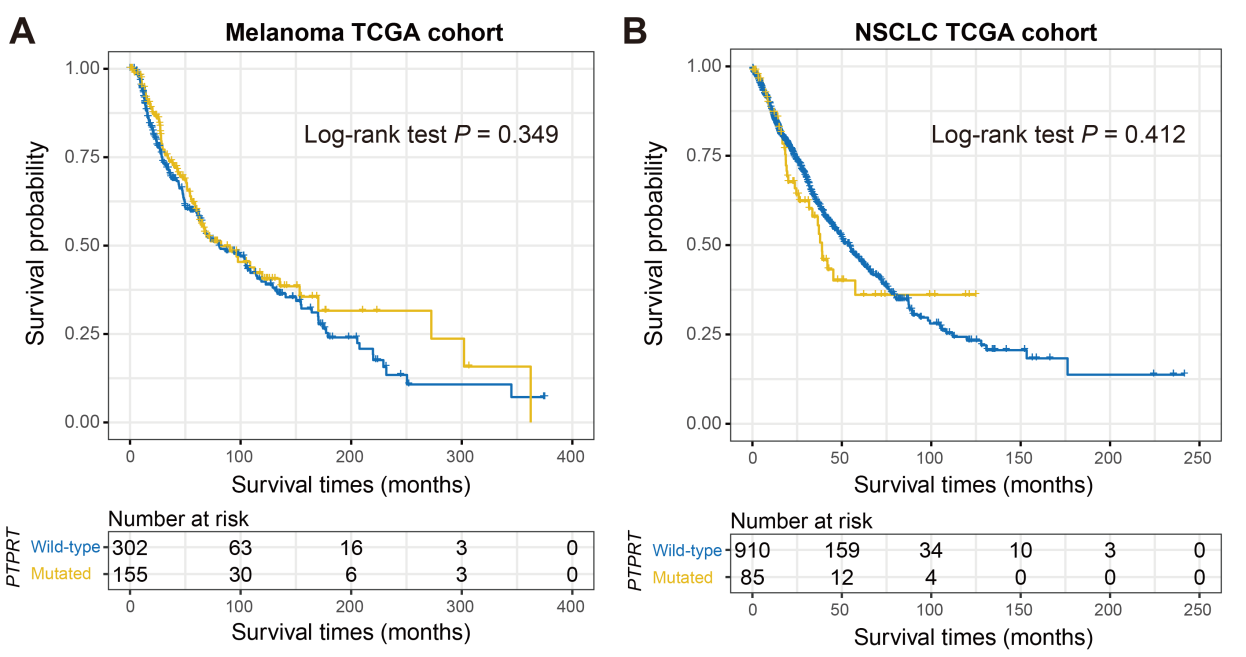

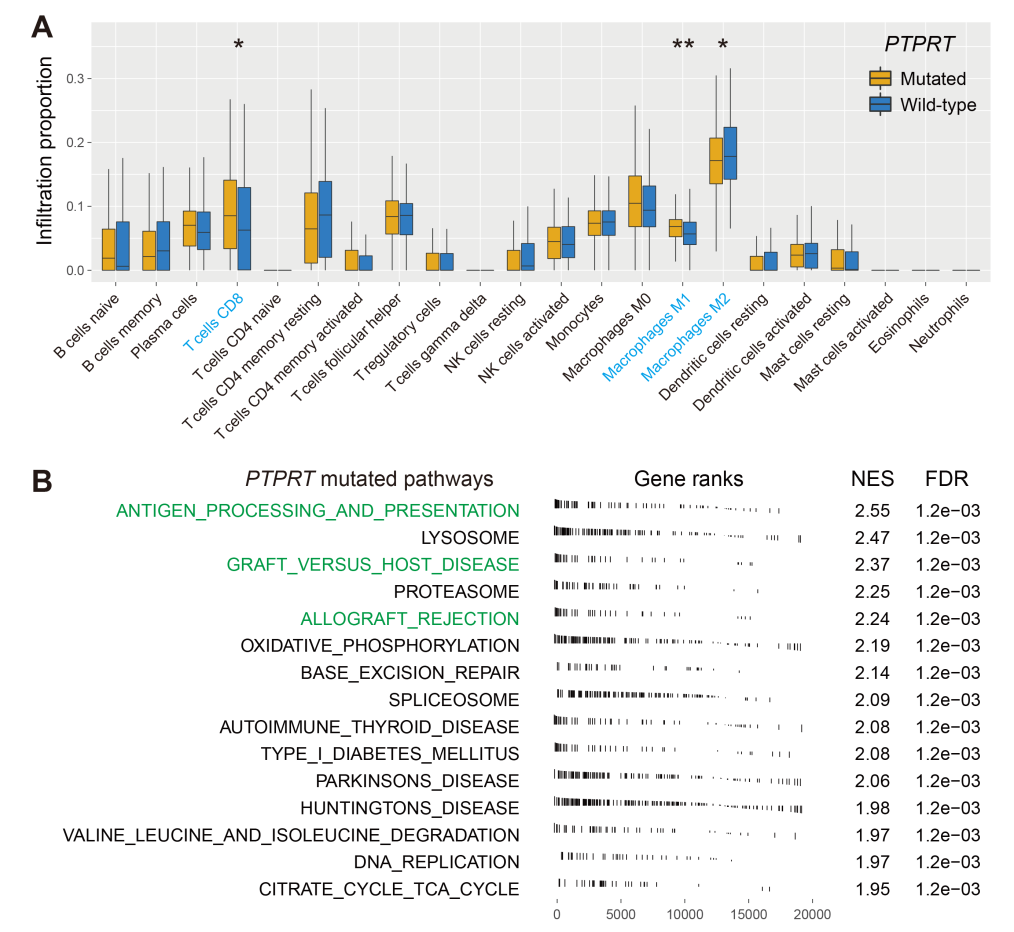
Figure S8.** Immune infiltration and GSEA analyses of *PTPRT* mutations in NSCLC. (A) Abundance of distinct immune infiltrated cells was evaluated with CIBERSORT algorithm according to *PTPRT* mutation status. Immune cells highlighted with blue were significantly differentially infiltrated between two groups. (B) GSEA results showed the top enriched pathways in *PTPRT* mutated NSCLC patients. Pathways highlighted with green were immune response-related.

**Figure S9.** Prognostic abilities of *PTPRT* mutations in (A) TCGA melanoma and (B) NSCLC cohorts.
